# Connecting Past Trauma With Current Mental Health Challenges: A Photovoice Study Exploring Men's Experiences

**DOI:** 10.1192/bjo.2024.204

**Published:** 2024-08-01

**Authors:** Sarah McKenzie

**Affiliations:** University of Otago, Wellington, New Zealand

## Abstract

**Aims:**

While evidence suggests men experience high rates of trauma, there is little qualitative research investigating men's experiences of past trauma and current mental health challenges. This study aimed to obtain a richer understanding of the trauma histories embedded in men's accounts of living with depression, anxiety, and suicidality, and how men responded to these challenges.

**Methods:**

Twenty-one New Zealand-based men were recruited from the community and asked to take photographs depicting their experiences of living with depression, anxiety, and suicidality including what had helped or hindered their recovery. Participants shared their narratives and photographs in semi-structured interviews.

**Results:**

The findings show an array of participant experiences of past trauma at the individual, family and community level. Three themes were inductively derived to describe how men responded to these traumas: (1) *struggling to survive* which describes the isolation and emotional pain of men's ever-present trauma, heightened by engaging in risky coping strategies; (2) *connecting with past trauma* referred to the participants' understanding of their trauma, disclosure (or not) and help-seeking; and (3) *moving forward* detailed the strategies employed by participants to overcome these challenging experiences and mend and sustain their mental health.

**Conclusion:**

The findings reinforce the importance of in-depth qualitative work towards revealing the impact of past trauma on men's current mental health as well as how men make sense of, disclose and cope with experiences of trauma. These findings have important implications for mental health practitioners working with men. Addressing trauma histories in men seeking help for current mental health challenges may play a key role in improving mental health services and interventions for men.

